# Supplementary material for: Sex differences in the risk profiles for anemia in people living with HIV, A cross sectional study
Source: PLoS One. 2025 Mar 10;20(3):e0319611. doi: 10.1371/journal.pone.0319611 (PMC11892841; doi:10.1371/journal.pone.0319611)
Supplement: Attachment — Submitted filename: PONE-D-24-48707.pdf [file pone.0319611.s003.pdf]

PLOS ONE

Sex Differences in the Risk Profiles for Anaemia in People Living with HIV, A Cross Sectional Study

--Manuscript Draft--

Manuscript Number:	PONE-D-24-48707
Article Type:	Research Article
Full Title:	Sex Differences in the Risk Profiles for Anaemia in People Living with HIV, A Cross Sectional Study
Short Title:	Sex Differences in Anaemia among PLWH
Corresponding Author:	Kingsley Kamvuma<br>Mulungushi University<br>Livingstone, Southern ZAMBIA
Keywords:	Anaemia; HIV; antiretroviral therapy; sex differences; PLWH; Zambia
Abstract:	Background<br><br>Anaemia in people living with HIV (PLWH) significantly impacts quality of life and health outcomes. This study aimed to determine sex differences in factors associated with anaemia in PLWH at Livingstone University Teaching Hospital, Zambia.<br><br>Methods<br><br>This cross-sectional study involved 631 PLWH aged 18 years or older who had been on combinational ART for at least 6 months. Data was collected via standardized questionnaires and medical records. Anaemia was defined as haemoglobin levels <13 g/dL for men and <12 g/dL for women, based on WHO criteria. Logistic regression models assessed the associated factors, stratified by sex.<br><br>Results<br><br>Participants had a median age of 44 years, with a female preponderance of 64.2%. The overall prevalence of anaemia was 36%, significantly higher in females (41.1%) compared to males (27.2%) (p<0.001). In females, waist circumference (AOR = 0.97, 95% CI: 0.95-0.99, P = 0.018), albumin levels (AOR = 0.96, 95% CI: 0.92-0.99, P = 0.047), NNRTI regimens (AOR = 2.78, 95% CI: 1.34-5.78, P = 0.006), microcytosis (AOR = 3.18, 95% CI: 1.26-8.03, P = 0.014), and hypertension (OR = 0.34, 95% CI: 0.13-0.87, P=0.024) were linked to anaemia in adjusted analysis but these associations were abrogated by males sex.<br><br>Conclusions<br><br>We found a 36% prevalence of anaemia among PLWH, with a higher prevalence in females (41%) compared to males (27%), including severe forms of anaemia. Among females, anaemia was linked to lower waist circumference, lower albumin levels, NNRTI regimens, microcytosis, and blood pressure but not males. Further studies are warranted.
Order of Authors:	Kingsley Kamvuma
	Benson M Hamooya
	Kaseya O.R. Chiyenu
	Yusuf Uthman Ademola
	Steward Mudenda
	Alfred Machiko
	Sepiso K Masenga
	Sody M. Munsaka

Powered by Editorial Manager® and ProduXion Manager® from Aries Systems Corporation

Additional Information:	
Question	Response
**Financial Disclosure**<br><br>Enter a financial disclosure statement that describes the sources of funding for the work included in this submission. Review the submission guidelines for detailed requirements. View published research articles from *PLOS ONE* for specific examples.<br><br>This statement is required for submission and **will appear in the published article** if the submission is accepted. Please make sure it is accurate.<br><br>**Funded studies**<br>Enter a statement with the following details:<br>• Initials of the authors who received each award<br>• Grant numbers awarded to each author<br>• The full name of each funder<br>• URL of each funder website<br>• Did the sponsors or funders play any role in the study design, data collection and analysis, decision to publish, or preparation of the manuscript?<br><br>Did you receive funding for this work?	The author(s) received no specific funding for this work.
**Competing Interests**<br><br>Use the instructions below to enter a competing interest statement for this submission. On behalf of all authors, disclose any competing interests that could be perceived to bias this work—acknowledging all financial support and any other relevant financial or non-financial competing interests.<br><br>This statement is required for submission and **will appear in the published article** if the submission is accepted. Please make sure it is accurate and that any funding sources listed in your Funding Information	The authors have declared that no competing interests exist.

Powered by Editorial Manager® and ProduXion Manager® from Aries Systems Corporation

later in the submission form are also declared in your Financial Disclosure statement.<br><br>View published research articles from *PLOS ONE* for specific examples.<br><br>**NO authors have competing interests**<br><br>Enter: *The authors have declared that no competing interests exist.*<br><br>**Authors with competing interests**<br><br>Enter competing interest details beginning with this statement:<br><br>*I have read the journal's policy and the authors of this manuscript have the following competing interests: [insert competing interests here]*<br><br>* typeset	
**Ethics Statement**<br><br>Enter an ethics statement for this submission. This statement is required if the study involved:<br><br>• Human participants<br>• Human specimens or tissue<br>• Vertebrate animals or cephalopods<br>• Vertebrate embryos or tissues<br>• Field research<br><br>Write "N/A" if the submission does not require an ethics statement.<br><br>General guidance is provided below. Consult the submission guidelines for detailed instructions. **Make sure that all information entered here is included in the Methods section of the manuscript.**	This study was approved by the University of Zambia Biomedical Research Ethics Committee (UNZABREC- REF. NO. 4062-2023) and all participants provided written informed consent. The study was conducted in accordance with the Declaration of Helsinki and Good Clinical Practice guidelines.

**Format for specific study types**<br><br>**Human Subject Research (involving human participants and/or tissue)**<br>• Give the name of the institutional review board or ethics committee that approved the study<br>• Include the approval number and/or a statement indicating approval of this research<br>• Indicate the form of consent obtained (written/oral) or the reason that consent was not obtained (e.g. the data were analyzed anonymously)<br><br>**Animal Research (involving vertebrate animals, embryos or tissues)**<br>• Provide the name of the Institutional Animal Care and Use Committee (IACUC) or other relevant ethics board that reviewed the study protocol, and indicate whether they approved this research or granted a formal waiver of ethical approval<br>• Include an approval number if one was obtained<br>• If the study involved *non-human primates*, add *additional details* about animal welfare and steps taken to ameliorate suffering<br>• If anesthesia, euthanasia, or any kind of animal sacrifice is part of the study, include briefly which substances and/or methods were applied<br><br>**Field Research**<br><br>Include the following details if this study involves the collection of plant, animal, or other materials from a natural setting:<br>• Field permit number<br>• Name of the institution or relevant body that granted permission	
**Data Availability**<br><br>Authors are required to make all data underlying the findings described fully available, without restriction, and from the time of publication. PLOS allows rare exceptions to address legal and ethical concerns. See the PLOS Data Policy and FAQ for detailed information.	Yes - all data are fully available without restriction

Powered by Editorial Manager® and ProduXion Manager® from Aries Systems Corporation

A Data Availability Statement describing where the data can be found is required at submission. Your answers to this question constitute the Data Availability Statement and **will be published in the article**, if accepted.<br><br>**Important:** Stating 'data available on request from the author' is not sufficient. If your data are only available upon request, select 'No' for the first question and explain your exceptional situation in the text box.<br><br>Do the authors confirm that all data underlying the findings described in their manuscript are fully available without restriction?	
**Describe where the data may be found in full sentences. If you are copying our sample text, replace any instances of XXX with the appropriate details.**<br><br>• If the data are **held or will be held in a public repository**, include URLs, accession numbers or DOIs. If this information will only be available after acceptance, indicate this by ticking the box below. For example: *All XXX files are available from the XXX database (accession number(s) XXX, XXX.*).<br>• If the data are all contained **within the manuscript and/or Supporting Information files**, enter the following: *All relevant data are within the manuscript and its Supporting Information files.*<br>• If neither of these applies but you are able to provide **details of access elsewhere**, with or without limitations, please do so. For example:<br><br>*Data cannot be shared publicly because of [XXX]. Data are available from the XXX Institutional Data Access / Ethics Committee (contact via XXX) for researchers who meet the criteria for access to confidential data.*<br><br>*The data underlying the results presented in the study are available from (include the name of the third party*	All relevant data are within the manuscript and its Supporting Information files.

Powered by Editorial Manager® and ProduXion Manager® from Aries Systems Corporation

and contact information or URL).<br>• This text is appropriate if the data are owned by a third party and authors do not have permission to share the data.<br><br>* typeset	
Additional data availability information:	Tick here if your circumstances are not covered by the questions above and you need the journal’s help to make your data available.

Powered by Editorial Manager® and ProduXion Manager® from Aries Systems Corporation

Manuscript

Click here to access/download;Manuscript;sex diff article plos one.docx docx.docx

Sex Differences in the Risk Profiles for Anaemia in People Living with HIV, A Cross Sectional Study

Kingsley Kamvuma[1,3], , Benson M Hamooya[2], Kaseya O.R. Chiyenu[5], Yusuf Uthman Ademola[1], Steward Mudenda[4], Alfred Machiko[6], Sepiso K Masenga[1] and Sody M. Munsaka[3]

[1]HAND Research Group, Mulungush University, School of Medicine and Health Sciences, Department of Pathology and Microbiology, Livingstone campus, Livingstone Zambia.

[2]HAND Research Group, Mulungush University, School of Medicine and Health Sciences, Department of Public Health, Livingstone campus, Livingstone Zambia.

[3]University of Zambia, School of Health Sciences, Department of Biomedical Sciences, Lusaka, Zambia.

[4]University of Zambia Department of Pharmacy, School of Health Sciences, Lusaka, Zambia

[5]Livingsotne University Teaching Hospital, Department of Internal Medicine, Livingstone, Zambia

[6]Cobberbelt Unversity, School Medicine, Department of Basic Science, Ndola, Zambia

*Corresponding author: Kingsley Kamvuma, Mulungushi University School of Medicine and Health Sciences, Department of Pathology and Microbiology, Livingstone Zambia. kamvumak@yahoo.com

Abstract

Background: Anaemia in people living with HIV (PLWH) significantly impacts quality of life and health outcomes. This study aimed to determine sex differences in factors associated with anaemia in PLWH at Livingstone University Teaching Hospital, Zambia.

Methods: This cross-sectional study involved 631 PLWH aged 18 years or older who had been on combinational ART for at least 6 months. Data was collected via standardized questionnaires and medical records. Anaemia was defined as haemoglobin levels <13 g/dL for men and <12 g/dL for women, based on WHO criteria. Logistic regression models assessed the associated factors, stratified by sex.

Results: Participants had a median age of 44 years, with a female preponderance of 64.2%. The overall prevalence of anaemia was 36%, significantly higher in females (41.1%) compared to males (27.2%) (p<0.001). In females, waist circumference (AOR = 0.97, 95% CI: 0.95-0.99, P = 0.018), albumin levels (AOR = 0.96, 95% CI: 0.92-0.99, P = 0.047), NNRTI regimens (AOR = 2.78, 95% CI: 1.34-5.78, P = 0.006), microcytosis

(AOR = 3.18, 95% CI: 1.26-8.03, P = 0.014), and hypertension (OR = 0.34, 95% CI: 0.13-0.87, P=0.024) were linked to anaemia in adjusted analysis but these associations were abrogated by males sex.

Conclusions: We found a 36% prevalence of anaemia among PLWH, with a higher prevalence in females (41%) compared to males (27%), including severe forms of anaemia. Among females, anaemia was linked to lower waist circumference, lower albumin levels, NNRTI regimens, microcytosis, and blood pressure but not males. Further studies are warranted.

Keywords: Anaemia, HIV, antiretroviral therapy, sex differences, PLWH, Zambia.

Introduction

Anaemia is a common comorbidity in people living with HIV (PLWH), significantly impacting their quality of life and clinical outcomes [1]. A recent meta-analysis revealed that the global pooled prevalence of anaemia in adults for both men and non-pregnant women is approximately 46.6% [2]. However, the prevalence of anaemia in PLWH is disproportionately higher in sub-Saharan Africa (SSA), particularly in Southern Africa (58 to 70%) [3], [4], [5], [6], [7], [8]. This indicates that despite advancements in combinational antiretroviral therapy (ART), anaemia remains prevalent in this population.

Anaemia PLWH can exacerbate HIV-related complications, such as immunosuppression, opportunistic infection, and poor treatment outcomes including disease progression and death [9], [10], [11]. Anamia in PLWH maybe a marker of underlying comorbidities, such as nutritional deficiencies, chronic inflammation, and other chronic diseases [12], [13]. It can also worsen quality of life, leading to fatigue, weakness, and decreased productivity [14]. Therefore, there is a need to conduct routine screening of anaemia and risk factors associated with anaemia among PLWH [3].

Existing studies have largely focused on the general population, neglecting the unique needs and sex specific characteristics of PLWH [2], [9]. While it is known that females may be at a higher risk of developing anaemia due to various biological factors [14], [15], and that men may also experience anaemia due varous factors non specific factors, there is still little or no information available about sex differences among PLWH [16]. By elucidating these differences, our study aimed to inform more effective, gender-sensitive clinical practices and public health strategies to mitigate anaemia.

This study aims to provide an understanding of the sex specific risk profiles for anaemia among PLWH. The overall goal of the study was to identify sex-specific factors associated with anaemia in PLWH attending an HIV clinic at Livingstone University Teaching Hospital (LUTH) in Zambia.

Methods

Study population and eligibility

This cross-sectional study was conducted among PLWH attending an ART clinic at LUTH, an urban tertiary care centre. Participants were recruited between 25th August 2023 and 30th December 2023. Following informed consent, we recruited adult PLWH aged 18years and above who had been receiving ART for ≥ 6 months. Pregnant women, participants with excessive menstrual bleeding and disorders of haemoglobin synthesis including Sickle cell anaemia and thalassaemia or malignant neoplasm were excluded.

Data collection

Sociodemographic and clinical data were collected using a standardized questionnaire. We also obtained data from the ART registry and the laboratory information system. The questionnaire covered demographic details (age, gender, marital status, and education level), HIV-related specifics (duration of ART, ART regimen and viral load), presence of comorbidities (hypertension and hepatitis B), and lifestyle factors (Excercise habits). Waist circumference was measured using a flexible tape measure positioned at the midpoint between the iliac crest and the lower rib margin, with measurements recorded to the nearest 0.1 cm.

We also collected laboratory samples for measurements of various parameters including viral load samples, full blood counts in ethylenediaminetetraacetic acid (EDTA) containers; a Becton Dickson flow cytometer was used to analyse CD4 counts and the viral load was analysed using Ampliprep/Taqman 96 PCR analyser in the laboratories at LUTH. Biochemical analyses were done on a Pentra C200 and HumaStar 80 clinical chemistry analyzer (Human Diagnostics, Germany) using kits supplied by the manufacturer.

Antiretroviral therapy regimens included non-nucleoside reverse transcriptase inhibitor (NNRTI) with options for efavirenz (EFV) or nevirapine (NVP) paired with abacavir/lamivudine or emtricitabine (ABC/3TC or FTC) or tenofovir disoproxil fumarate/emtricitabine (TDF/FTC). Protease inhibitor (PI) regimens included lopinavir/ritonavir (LPV/r) or atazanavir/ritonavir (ATV/r) combined with ABC/3TC or

FTC, AZT/FTC, or TDF/FTC. Integrase strand transfer inhibitor (INSTI) regimens included dolutegravir (DTG) with TDF/3TC.

Sample size determination

The sample size calculation utilized the single population proportion formula, assuming a proportion of 50%. We set the desired margin of error at 0.05 and a confidence level of 99%. With a total population of 3,880 patients receiving antiretroviral therapy (ART) at LUTH, sample size was determined to be 578. To accommodate potential ineligibility after consent and non-response, a 10% contingency was added. Therefore, the final sample size was calculated to be 631 participants.

Operational Definitions

Viral suppression was defined according to the Zambia Consolidated Guidelines for Treatment and Prevention of HIV Infection as consistent adherence to antiretroviral therapy (ART) and achieving a viral load of less than 1,000 copies of HIV RNA per milliliter of blood [17]. The primary outcome anaemia was defined as haemoglobin <13 g/dL for men and <12 g/dL for women, according to the World Health Organization criteria [18]. Further subclassified the anaemia as mild (11–12.9 g/dl for males and 11–11.9 g/dl for females), moderate (8–10.9 /dl) and severe (<7.9 g/dl) [18]. The type of anaemia s was assessed using mean cell volume (MCV) values, categorizing them as microcytosis (<80 fL), normocytosis (80-100 fL), and macrocytosis (>100 fL) [19]

Statistical Analysis

For our statistical analyses, we employed SPSS software (version 22). Categorical data were summarized using frequencies and proportions. Statistical significance for continuous variables was assessed using Two-way ANOVA with Fisher's Least Significant Difference multiple comparison test (*p<0.05; **p<0.01, ***p<0.001; ns=p>0.05). We further conducted logistic regression (both univariable and multivariable) to estimate factors associated with anaemia, stratified by sex. The covariates included in the final models were selected based on published evidence and variables that demonstrated statistical significance in the univariable analysis. Additionally, we assessed model fitness using the Hosmer-Lemeshow goodness-of-fit test, with statistical significance defined as $P<0.05$.

Ethical Approval

This study was approved by the University of Zambia Biomedical Research Ethics Committee (UNZABREC- REF. NO. 4062-2023) and all participants provided written informed consent. The study was conducted in accordance with the Declaration of Helsinki and Good Clinical Practice guidelines.

Reporting format

We adhered to the strengthening the reporting of observational studies in epidemiology guidelines for reporting observational studies. See Supplementary 1 file.

RESULTS

Background Characteristics

We studied 631 PLWH with a median age of 44 years and 63.2% female predominance. We found the prevalence of anaemia to be 36% overall but was higher in females compared to males (41% vs 27%, p<0.001) (Fig. 1). Among females The majority of participants were virally suppressed (81.6%) and were on NNRTI based regimen (67.2%) (Table 1). Among females, 21.1% had mild anaemia , 15% had moderate anaemia , and 5% had severe anaemia . In contrast, 21.1% of males had mild anaemia , 5.6% had moderate anaemia , and only 0.4% had severe anaemia (Fig. 1).

Fig. 1: Sex differences in prevalence of anaemia and its severity. Females exhibit a higher overall prevalence of anaemia (41.1%) compared to males (27.1%). In terms of severity**,** both sexes have equal prevalence of mild anaemia (21.1%). However, moderate anaemia is more common in females (15%) than in males (5.6%), and severe anaemia is significantly higher in females (5%) compared to males (0.4%).

Relationship between anaemia and Sex specitic characteristics in PLWH

We found that male participants on NNRTI regimens were more likely to have anaemia compared to those on INSTI or PI regimens (p=0.03), (Table 1). Similarly, females on NNRTI regimens were more likely to be anaemic compared to those on INSTI or PI regimens (p=0.008). Hypertensive females were less likely to be anaemic (25% vs 43.3%, P=0.011), but this relationship was not observed in males. Based on the Mean Cell Volume (MCV), there were no significant differences across MCV categories in females. However, 57% of anaemic females had microcytosis, compared to 39% with normocytosis and 39% with macrocytosis (p=0.055). In contrast, anaemic males were more likely to have macrocytosis (40%) compared to normocytosis (24%) and microcytosis (16%) (p=0.035) (Table 1).

Table 1: Shows Sex specific characteristics stratified by anaemia status

Background characteristics	Total	MALES Anaemia 63 (27%)	MALES No anaemia 169 (73%)	p	FEMALES Anaemia 164 (41.1)	FEMALES No anaemia 235(58.9)	p <0.001
Physical Excerise				0.229			0.834
Yes	189	21(22.8)	71(77.2)		39 (40.2)	58(59.8)	
no	437	42(30.0)	98(70.0)		123 (41.4)	174(58.6)	
Marital status				0.683			0.535
Yes	326	43(26.4)	120 (73.6)		64 (39.3)	99 (60.7)	
No	305	20(28.9)	49(71.0)		100(42.4)	136(57.6)	
ART regimen				**0.03**			**0.008**
INSTI	132	8(14.0)	49(86.0)		20 (26.7)	55(73.3)	
NNRTI	424	47(32.4)	98(67.6)		128(45.9)	151(54.1)	
PI	75	8(26.7)	22(73.3)		16(35.6)	29(64.4)	
Duration on ART mo, m(IQR)	631	120 (48, 144)	108(64,149)	0.919	108(60, 144)	108(60, 144)	0.492
HBsAg				0.350			0.657
Yes	42	4(40)	6(60)		12(37.5)	20(62.5)	
No	588	59(26.6)	163(73.4)		152(41.5)	214(58.5)	
Viral load				0.748			0.451
Suppressed	514	51 (26.4)	142 (73.6)		129 (40.2)	192 (59.8)	
Unsuppressed	116	11 (28.9)	27 (71.1)		35 (44.9)	43 (55.1)	
CD4+ Count				0.150			0.168
<200	35	3 (23.1)	10 (76.9)		12 (54.5)	10 (45.5)	
201-350	49	4 (12.9)	27 (87.1)		18 (32.1)	38 (68.9)	
>351	505	55 (29.4)	132 (70.6)		133 (41.8)	185 (58.2)	
Hypertension				0.125			**0.011**
Yes	79	4 (14.8)	23 (85.2)		13 (25)	39 (75)	
No	552	59 (29.2)	146 (70.8)		151 (43.3)	196 (56.7)	
MCV				**0.035**			0.055
Microcytosis	77	4 (16.7)	20 (83.3)		30 (56.6)	23 (43.4)	
Normocytosis	397	36 (24)	114 (76)		97 (39.3)	150 (60.7)	
Macrocytosis	148	22 (40)	33 (60)		36 (38.7)	57 (61.3)	

Abbreviations: m (IQR): Mean (Interquartile Range), BMI: Body Mass Index, WC: Waist Circumference, ALT: Alanine Transaminase, AST: Aspartate Transaminase, MCV: Mean Cell Volume, WBC: White Blood Cells, GFR: Glomerular Filtration Rate, ART: Antiretroviral Therapy, INSTI: Integrase Strand Transfer Inhibitors, NNRTI: Non-Nucleoside Reverse Transcriptase Inhibitors, PI: Protease Inhibitors

Females with anaemia were significantly younger than their male counterparts (mean age 44.0 ± 11.5 vs. 48.5 ± 15.9 years, $p < 0.05$) (Figure 2A). Both males and females with anaemia had lower body mass index (BMI) compared to those without anaemia (males: 20.7 ± 3.5 vs. 22.2 ± 5.1 kg/m²; females: 23.9 ± 5.8 vs. 24.5 ± 5.1 kg/m², $p < 0.0001$) (Figure 2B). Similarly, males with anaemia exhibited a lower waist circumference than females, was particularly more pronounced in those with anaemia (males: 75.6 ± 11.3 vs. 80.3 ± 12.8 kg/m²; females: 81.1 ± 11.1 vs. 83.9 ± 12.9 kg/m², $p < 0.001$) (Figure 2C). Furthermore, females with anaemia had been on ART longer than those without anaemia (124 ± 151 vs. 103 ± 50.1 months, $p<0.05$) Figure 2D. Alanine aminotransferase levels were higher in males with anaemia compared to females with anaemia (24.8 ± 27.1 vs. 20.3 ± 10.4 mmol/l, $p < 0.05$) (Figure 2E). We did not find statistical sex differences between the anaemic and non anaemic groups in (Fig. 2F) aspartate aminotransferase levels, (Fig. 2G) asolute WBC count, (Fig. 2H) neutrophil count, (Fig. 2I) lymphocyte counts. Monocyte counts showed sex-specific differences, being lower in females with anaemia but higher in females without anaemia compared to males with or without anaemia (males: 0.4 ± 0.2 vs. 0.5 ± 0.8 x10⁹/L vs female: 0.1 ± 0.01 vs. 0.3 ± 0.1 x10⁹/L, $p < 0.01$) (Figure 2J). Platelet counts were higher in females with anaemia compared to females without anaemia (276.0 ± 80 vs. 257.6 ± 80 x10⁹/L, $p < 0.05$) (Figure 2K). We also did not find statically significant differences creatinine (Fig.2L) and eGFR values (Fig. 2M) although females without anaemia had lower values (males: 113 ± 19 vs 113 ± 21 vs female 107±20 vs 101±20) and urea levels (Fig. 2N). Lastly, albumin levels were lower in both males and females with anaemia compared to those without anaemia (males: 39.8 ± 6.2 vs. 42.6 ± 7.9 kg/m²; females: 40.4 ± 6.8 vs. 42.0 ± 7.1 kg/m², $p < 0.05$) (Figure 2O).

Fig 2. Sex differences and comparison of characteristics in the study.) Shows differences in males and females with anaemia compared to those without. (A) Age, (B) Body mass index, (C) waist circumference, (D) Duration on ART, (E) Alanine aminotransferase levels, (F) Aspatate aminotransferase, (G) White blood Counts counts, (H)neutrophil counts, (I) lymphocyte counts, (J)monocytes, (K)platelets, (L)creatinine, (M) eGFR, (N)urea, (O) albumin exhibit variations between groups. Statistical significance was assessed using Two-way ANOVA with Fisher's LSD multiple comparison test (*p<0.05; **p<0.01, ***p<0.001; ns=p>0.05).

Factors associated with anaemia in females

In the unadjusted analysis, several factors were significantly associated with anaemia in females, (Table 2). The odds of anaemia decreased by 2% with each additional year in age (OR = 0.98, 95% CI: 0.96-0.99, P = 0.03). Lower waist circumference was linked to a 2% increase in the odds of anaemia (OR = 0.98, 95% CI: 0.96-0.99, P = 0.024). Increased monocyte levels were associated with a 71% decrease in the odds of anaemia (OR = 0.29, 95% CI: 0.09-0.96, P = 0.043). Higher albumin levels were associated with a 4% decrease in the odds of anaemia (OR = 0.96, 95% CI: 0.92-0.99, P = 0.036). Females on NNRTI regimens had a 133% increase in the odds of anaemia compared to those on INSTI regimens (OR = 2.33, 95% CI: 1.32-4.09, P = 0.003). Microcytosis was associated with a 107% increase in the odds of anaemia (OR = 2.07, 95% CI: 1.04-4.09, P = 0.038). Lastly, hypertension was associated with a 57% decrease in the odds of anaemia (OR = 0.43, 95% CI: 0.22-0.84, P = 0.013).

In the adjusted analysis, lower waist circumference was associated with a 3% increase in the odds of anaemia (OR = 0.97, 95% CI: 0.95-0.99, P = 0.018), Table 2. Higher albumin levels were linked to a 4% decrease in the odds of anaemia (OR = 0.96, 95% CI: 0.92-0.99, P = 0.047). Females on NNRTI regimens had a 2.78 times higher odds of anaemia compared to those on INSTI regimens (OR = 2.78, 95% CI: 1.33-5.82, P = 0.006). Microcytosis was associated with a 3.18 times higher odds of anaemia (OR = 3.18, 95% CI: 1.27-7.99, P = 0.014). Lastly, hypertension was associated with a 66% decrease in the odds of anaemia (OR = 0.34, 95% CI: 0.14-0.87, P = 0.024).

Table 2: Logistic regression analysis of factors associated with anaemia in Females

Variables	Unadjusted analysis		Adjusted Analysis	
	OR (95% CI)	*P*	AOR (95% CI	*P*
Age (years)	0.98 (0.96,0.99)	**0.03**	0.99 (0.97 -1.02)	0.885
Bmi kg/m2	0.98 (0.94,1.02)	0.258		
WC (cm)	0.98 (0.96,0.99)	**0.024**	0.97 (0.95-0.99)	**0.018**
Duration on ART mo, m(IQR)	1.00 (0.99,1.00)	0.08	1.001 (0.99-1.01)	0.402

Art regimen				
INSTI	1		1	
NNRTI	2.33(1.32,4.09)	**0.003**	2.78 (1.33-5.82)	**0.006**
PI	1.52(0.68, 3.37)	0.305	2.58 (0.89-7.46)	0.08
Mean cell Volume				
Macrocytosis	1		1	
Microcytosis	2.07 (1.04-4.09)	**0.038**	3.18 (1.27-7.99)	**0.014**
Normocytosis	1.02 (0.63-1.67)	0.925	1.59 (0.81-3.13)	0.183
CD4+ Count				
>350	1		1	
<200)	1.67 (0.70-3.98)	0.247	3.08 (0.92-10.28)	0.068
201-350	0.66 (0.36-1.21	0.175	0.45 (0.2-1.03)	0.060
HTN, mmHg				
No	1		1	
Yes	0.43 (0.22-0.84)	**0.013**	0.34 (0.14-0.87)	**0.024**
Viral Load				
Suppressed	1		1	
Unsuppressed	*0.83 (0.50-1.36)*	0.451	1.03 (0.52-2.04)	0.943
Lymphocytes 10^9/L	0.97 (0.72, 1.30)	0.847		
Monocytes 10^9/L	0.29 (0.09, 0.96)	**0.043**	0.59 (0.15-2.27)	0.441
Creatinine µmol/l	0.99 (0.99, 1.01)	0.841		
Albumin g/dl	0.96 (0.92, 0.99)	**0.036**	0.96 (0.92-0.99)	**0.047**

Abbreviations: BMI Body Mass Index, WC Waist Circumference, mo months, cm centre meters, mmHg millimetres of mercury, ART Antiretroviral therapy, CD4 cluster of differentiation 4, HTN Hypertension, INSTI: Integrase Strand Transfer Inhibitors, NNRTI: Non-Nucleoside Reverse Transcriptase Inhibitors, PI: Protease Inhibitors

Factors Associated with Anaemia in Males

In the unadjusted analysis, several factors were significantly associated with anaemia in men (Table 3). A higher BMI was associated with an 8% decrease in the odds of anaemia (OR = 0.92, 95% CI: 0.85-0.99, P = 0.034). Lower waist circumference was associated with a 3% increase in the odds of anaemia (OR = 0.97, 95% CI: 0.95-0.99, P = 0.014). Men on NNRTI regimens had a 194% increase in the odds of anaemia compared to those on INSTI regimens (OR = 2.94, 95% CI: 1.29-6.70, P = 0.01). Higher creatinine levels were associated with a 1% decrease in the odds of anaemia (OR = 0.99, 95% CI: 0.96-

0.99, P = 0.014). Higher albumin levels were associated with a 5% decrease in the odds of anaemia (OR = 0.95, 95% CI: 0.91-0.99, P = 0.019).

In the adjusted analysis, no variables remained significantly associated with anaemia in men.

Table 3: Logistic regression analysis of factors associated with anaemia in Males

Variables	Unadjusted analysis		Adjusted Analysis	
	OR (95% CI)	Pvalue	AOR (95% CI)	pvalue
Age (years)	1.02 (0.99,1.04)	0.088	1.01 (0.97-1.05)	0.656
Bmi kg/m^2	0.92 (0.85,0.99)	**0.034**	0.97 (0.85-1.11)	0.666
Waist circumference (cm)	0.97 (0.95, .99)	**0.014**	0.97 (0.93-1.01)	0.111
Duration on ART mo, m(IQR)	1.00 (0.99, 1.00)	0.391		
ART regimen				
INSTI	1			
NNRTI	2.94 (1.29,6.70)	**0.01**	2.59 (0.59-11.46)	0.21
PI	2.23 (0.74, 6.70)	0.154	2.53 (0.39 -16.08)	0.324
Types of Anaemia				
Microcytosis	0.30 (0.09-0.99)	0.050	0.31 (0.04-2.46)	0.268
Normocytic	0.47 (0.24-0.91)	**0.026**	0.64 (0.21-1.93)	0.426
CD4+ count				
>350	1			0.411
201-350	0.36 (0.12-1.06)	0.064	0.38 (0.07-2.09)	0.267
<200	0.72 (0.19-2.72	0.628	0.34 (0.03-4.19)	0.401
Lymphocytes 10^9/L	1.34 (0.90, 1.98)	0.147		
Monocytes 10^9/L	0.58 (0.17, 1.99)	0.39		
Creatinine µmol/l	0.99 (0.96, 0.99)	**0.014**	0.98 (0.95-1.01)	0.114
Urea mg/dl	0.72 (0.55-0.94)	**0.016**	0.77 (0.47-1.26)	0.296
Albumin g/dl	0.95 (0.91, 0.99)	**0.019**	1.03 (0.96-1.09)	0.479

Abbreviations: BMI Body Mass Index, Alt Alanine Aminotransferase, WBC White cell count, mo months, cm centre meters, mmHg millimetres of mercury, HTN HypertensionART Antiretroviral therapy

Discussion

People Living with HIV (PLWH) who are on combinational ART still experience anaemia, but the factors contributing to its persistence are not well understood in Zambia. In this study, we examined the sex differences in factors associated with anaemia in an adult population of 631 PLWH in Sub-Saharan Africa, Zambia. We found a significantly higher

prevalence of anaemia in females compared to males. Among females, waist circumference, albumin levels, being an NNRTI based regimen and microcytosis was also associated with anaemia in this cohort but this relationship was abrogated by male sex as no variable remained statistically significant on multivariable analysis. Our findings suggest the need to closely monitor anaemia among PLWH, especially among females.

Our study found an overall prevalence of anaemia among PLWH of 36%, which is higher than the 16.8% reported in Uganda [20], 25.35% in East Africa [21], and 34% in Ethiopia [22] but lower than that reported in other studies including Indonesia 40.3%[23], China 55.15%[24], Nepal 66.7%[25]. Additioanlly, a global systematic review and meta-analysis found a prevalence of 46.6% for adults PLWH[2]. Our findings and those reported in other studies demonstrate the need to effectively monitor the haemoglobin levels of PLWH.

The sex differences observed may be due to several factors: biological differences such as menstrual blood loss and hormonal influences, greater nutritional deficiencies among women in low-resource settings, socio-economic barriers limiting women's access to healthcare and nutrition [26], [27]. Among females, microcytosis is linked to iron deficiency due to menstrual blood loss, exacerbated by chronic inflammation of HIV [28].

Although the exact mechanisms are not fully understood, the association of Anaemia with lower blood pressure maybe explained by several biological mechnisms that have been suggested. Anaemia reduces the free hemoglobin's binding to nitric oxide, leading to vasodilation and lower blood pressure [29], [30]. Additionally, previous reports have indicated that hemoglobin is strongly related to arterial stiffness, as measured by pulse wave velocity, which inversely anaemia predisposes these individuals to hypotension [31]. Lastly, chronic inflammation and immune activation in HIV infection can also lead to vasodilation and may impair iron availability for erythropoiesis, exacerbating anaemia [32].

Poor nutritional status indicated by lower waist circumference in our study, predisposes PLWH to anaemia [33]. Studies have shown that anaemia is associated waist circumference in HIV-infected women. Additionally, low albumin levels, associated with anaemia in people living with HIV, also reflect poor nutritional status and increased

catabolism [34]. Albumin, is also a negative acute-phase reactant, decreases during inflammatory states, further suppressing erythropoiesis [35]. Addressing nutritional deficiencies and managing inflammation are critical in mitigating anaemia in this population.

NNRTIs were associated with higher anaemia risk compared to INSTIs. The exact mechanisms underlying this association are not known but the impact of NNRTI maybe related to long term use, potentially leading bone toxicities and haematological complications, since most of our participants were initiated on NNRTIs [36]. Our findings differ from Harding et al., who reported a higher anaemia risk in PLWH on INSTI regimens compared to those on NNRTIs. [37].

The study's findings emphasize the necessity for sex-specific interventions to manage anaemia among PLWH in Sub-Saharan Africa. For females, interventions should focus on addressing nutritional suppport. Additionally, transitioning women from NNRTI-based regimens to alternatives like INSTIs when appropriate can reduce the risk of anaemia . Regular monitoring of hemoglobin levels, waist circumference, and albumin levels in women, along with comprehensive reproductive health services, will help alleviate anaemia and improve overall health outcomes.

Our study's limitations include the absence of direct measurements of markers of nutritional anaemia and inflammatory markers, as well as missing data on a few variables, potentially affecting our analysis. Despite this our study reveals key factors linked to anaemia in PLWH in Sub-Saharan Africa, emphasizing sex-specific differences

Conclusions

Our study revealed a higher prevalence of anaemia in females of 41% compared to males 27% in PLWH including the severe forms of anaemia i.e. moderate and severe anaemia. Associated factors differed by sex such that onlyblood pressure, waist circumference, microcytosis and NNRTI based regimen were associated with anaemia in females but this relationship was abrogated in males. These findings emphasize the importance of sex-specific strategies in managing anaemia among PLWH.

Competing interests:

The authors have declared that no competing interests exist.

Funding:

This work was supported by the Fogarty International Center and National Institute of Diabetes and Digestive and Kidney Diseases of the National Institutes of Health grants 2D43TW009744 (SKM), R21TW012635 (SKM) and the American Heart Association AwardNumber24IVPHA1297559https://doi.org/10.58275/AHA.24IVPHA1297559.pc.gr.193866 (SKM).

Author Contributions

K.K., S.K.M conceptualized the study and K.K., wrote the draft manuscript. S.M.M, B.M.H., W.C, AM, K.O.R.C, YUA and SM edited different sections of the manuscript. K.K, SKM, B.M.H, S.M.M analysed the data and created all the tables and figures, K.O.R.C,A.M, JAM, S.M. and S.K.M. finalized the manuscript. All authors have read and agreed to the published version of the manuscript.

Data availability

The datasets used during the current study is available from the corresponding author on reasonable request.

References

[1] P. Volberding, 'The Impact of Anaemia on Quality of Life in Human Immunodeficiency Virus—Infected Patients', *J. Infect. Dis.*, vol. 185, no. Supplement_2, pp. S110–S114, May 2002, doi: 10.1086/340198.

[2] G. Cao, Y. Wang, Y. Wu, W. Jing, J. Liu, and M. Liu, 'Prevalence of anaemia among people living with HIV: A systematic review and meta-analysis', *EClinicalMedicine*, vol. 44, p. 101283, Jan. 2022, doi: 10.1016/j.eclinm.2022.101283.

[3] T. A. Ageru, M. M. Koyra, K. D. Gidebo, and T. L. Abiso, 'Anaemia and its associated factors among adult people living with human immunodeficiency virus at Wolaita Sodo University teaching referral hospital', *PLoS ONE*, vol. 14, no. 10, p. e0221853, Oct. 2019, doi: 10.1371/journal.pone.0221853.

[4] I. Dobe, N. Manafe, N. Majid, I. Zimba, B. Manuel, and A. Mocumbi, ‘Patterns of cardiovascular risk and disease in HIV-positive adults on anti-retroviral therapy in Mozambique’, *Cardiovasc. J. Afr.*, vol. 31, no. 4, pp. 190–195, 2020, doi: 10.5830/CVJA-2020-007.

[5] A. E. Ezeamama *et al.*, ‘Evolution of Anaemia Types During Antiretroviral Therapy—Implications for Treatment Outcomes and Quality of Life Among HIV-Infected Adults’, *Nutrients*, vol. 11, no. 4, p. 755, Mar. 2019, doi: 10.3390/nu11040755.

[6] A. D. Kerkhoff, R. Wood, F. G. Cobelens, A. Gupta-Wright, L.-G. Bekker, and S. D. Lawn, ‘The predictive value of current haemoglobin levels for incident tuberculosis and/or mortality during long-term antiretroviral therapy in South Africa: a cohort study’, *BMC Med.*, vol. 13, p. 70, Apr. 2015, doi: 10.1186/s12916-015-0320-9.

[7] Z. Tamir, J. Alemu, and A. Tsegaye, ‘Anaemia among HIV Infected Individuals Taking ART with and without Zidovudine at Addis Ababa, Ethiopia’, *Ethiop. J. Health Sci.*, vol. 28, no. 1, p. 73, Jan. 2018, doi: 10.4314/ejhs.v28i1.9.

[8] M. Melku *et al.*, ‘Magnitude of anaemia and undernutrition among HIV-infected children who took HAART: a retrospective follow-up study’, *Am. J. Blood Res.*, vol. 10, no. 5, pp. 198–209, Oct. 2020.

[9] K. E. Gezae, K. Hagos, and A. A. Gebreslassie, ‘Severity and Determinants of Anaemia in TB/HIV Coinfected Adults at Mekelle, Ethiopia: Hospital Based Retrospective Study’, *J. Trop. Med.*, vol. 2023, p. e5555030, May 2023, doi: 10.1155/2023/5555030.

[10] M. Araújo-Pereira *et al.*, ‘Effect of the relationship between anaemia and systemic inflammation on the risk of incident tuberculosis and death in people with advanced HIV: a sub-analysis of the REMEMBER trial’, *eClinicalMedicine*, vol. 60, p. 102030, Jun. 2023, doi: 10.1016/j.eclinm.2023.102030.

[11] S. Takuva, M. Maskew, A. T. Brennan, I. Sanne, A. P. MacPhail, and M. P. Fox, ‘Anaemia among HIV-Infected Patients Initiating Antiretroviral Therapy in South Africa: Improvement in Hemoglobin regardless of Degree of Immunosuppression and the Initiating ART Regimen’, *J. Trop. Med.*, vol. 2013, p. e162950, Aug. 2013, doi: 10.1155/2013/162950.

[12] F. O. Demitto *et al.*, ‘Impact of Persistent Anaemia on Systemic Inflammation and Tuberculosis Outcomes in Persons Living With HIV’, *Front. Immunol.*, vol. 11, p. 588405, Sep. 2020, doi: 10.3389/fimmu.2020.588405.

[13] M. Araújo-Pereira *et al.*, ‘Relationship Between Anaemia and Systemic Inflammation in People Living With HIV and Tuberculosis: A Sub-Analysis of the CADIRIS Clinical Trial’, *Front. Immunol.*, vol. 13, p. 916216, Jun. 2022, doi: 10.3389/fimmu.2022.916216.

[14] P. N. Gona, C. M. Gona, V. Chikwasha, C. Haruzivishe, C. C. Mapoma, and S. R. Rao, ‘Intersection of HIV and Anaemia in women of reproductive age: a 10-year analysis of three Zimbabwe demographic health surveys, 2005–2015’, *BMC Public Health*, vol. 21, p. 41, Jan. 2021, doi: 10.1186/s12889-020-10033-8.

[15] B. N. Harding *et al.*, ‘Anaemia risk factors among people living with HIV across the United States in the current treatment era: a clinical cohort study’, *BMC Infect. Dis.*, vol. 20, no. 1, p. 238, Mar. 2020, doi: 10.1186/s12879-020-04958-z.

[16] A. Aemro, B. S. Workneh, E. G. Mekonen, M. Wassie, and B. Chekol, ‘Prevalence of anaemia and its associated factors among HIV-infected adults at the time of ART initiation at Debre Markos Comprehensive Specialized Hospital, Northwest Ethiopia: a retrospective cross-sectional study’, *BMJ Open*, vol. 12, no. 6, p. e057235, Jun. 2022, doi: 10.1136/bmjopen-2021-057235.

[17] ‘Zambia-Consolidated-Guidelines-for-Treatment-and-Prevention-of-HIV-Infection-2020.pdf’. Accessed: Jun. 27, 2024. [Online]. Available: https://www.moh.gov.zm/wp-content/uploads/filebase/Zambia-Consolidated-Guidelines-for-Treatment-and-Prevention-of-HIV-Infection-2020.pdf

[18] ‘Haemoglobin concentrations for the diagnosis of anaemia and assessment of severity’. Accessed: Mar. 28, 2024. [Online]. Available: https://www.who.int/publications-detail-redirect/WHO-NMH-NHD-MNM-11.1

[19] B. S. Maner and L. Moosavi, ‘Mean Corpuscular Volum’, 2023, Accessed: Jul. 13, 2024. [Online]. Available: https://www.ncbi.nlm.nih.gov/books/NBK545275

[20] R. Kaudha *et al.*, ‘Anaemia in HIV Patients Attending Highly Active Antiretroviral Therapy Clinic at Hoima Regional Referral Hospital: Prevalence, Morphological Classification, and Associated Factors’, *HIVAIDS Auckl. NZ*, vol. 15, pp. 621–632, Oct. 2023, doi: 10.2147/HIV.S425807.

[21] F. Getu, M. Aynalem, M. Walle, and B. Enawgaw, ‘The prevalence of anaemia among human immunodeficiency virus-infected individuals in East Africa: A systematic review and meta-analysis’, *Medicine (Baltimore)*, vol. 102, no. 20, p. e33810, May 2023, doi: 10.1097/MD.0000000000033810.

[22] K. W. Zerihun, G. A. Bikis, and E. A. Muhammad, ‘Prevalence and associated factors of anaemia among adult human immune deficiency virus positive patients on anti-retroviral therapy at Debre tabor Hospital, Northwest Ethiopia’, *BMC Res. Notes*, vol. 12, no. 1, p. 168, Mar. 2019, doi: 10.1186/s13104-019-4214-3.

[23] S. A. Aryastuti, S. R. Dewi, and S. Masyeni, ‘High Prevalence of Anaemia among Human Immunodeficiency Virus-Infected Patients: A Cross-sectional Study in Bali-Indonesia’, *Biomed. Pharmacol. J.*, vol. 14, no. 1, pp. 329–334, Mar. 2021.

[24] J. L. Lai *et al.*, ‘Prevalence and risk factors of anaemia in hospitalised HIV-infected patients in southeast China: a retrospective study’, *Epidemiol. Infect.*, vol. 147, p. e81, Jan. 2019, doi: 10.1017/S0950268818003618.

[25] S. K. Sah, P. Dahal, G. B. Tamang, D. K. Mandal, R. Shah, and S. B. Pun, ‘Prevalence and Predictors of Anaemia in HIV-Infected Persons in Nepal’, *HIVAIDS Auckl. NZ*, vol. 12, pp. 193–200, Jun. 2020, doi: 10.2147/HIV.S244618.

[26] S. Aziz Ali *et al.*, ‘Factors associated with anaemia among women of the reproductive age group in Thatta district: study protocol’, *Reprod. Health*, vol. 16, no. 1, p. 34, Mar. 2019, doi: 10.1186/s12978-019-0688-7.

[27] T. Gebreegziabher, N. Regassa, M. Wakefield, K. Pritchett, and S. Hawk, ‘Disparities in the prevalence and risk factors of anaemia among children aged 6–24 months and 25–59 months in Ethiopia’, *J. Nutr. Sci.*, vol. 9, p. e36, Jan. 2020, doi: 10.1017/jns.2020.29.

[28] C. Camaschella, A. Nai, and L. Silvestri, ‘Iron metabolism and iron disorders revisited in the hepcidin era’, *Haematologica*, vol. 105, no. 2, Art. no. 2, Feb. 2020, doi: 10.3324/haematol.2019.232124.

[29] C. C. Helms, M. T. Gladwin, and D. B. Kim-Shapiro, ‘Erythrocytes and Vascular Function: Oxygen and Nitric Oxide’, *Front. Physiol.*, vol. 9, Feb. 2018, doi: 10.3389/fphys.2018.00125.

[30] F. Atsma, I. Veldhuizen, W. de Kort, M. van Kraaij, P. Pasker-de Jong, and J. Deinum, ‘Hemoglobin Level Is Positively Associated With Blood Pressure in a Large Cohort of Healthy Individuals’, *Hypertension*, vol. 60, no. 4, pp. 936–941, Oct. 2012, doi: 10.1161/HYPERTENSIONAHA.112.193565.

[31] S.-H. Sung *et al.*, ‘Wave Reflections, Arterial Stiffness, and Orthostatic Hypotension’, *Am. J. Hypertens.*, vol. 27, no. 12, pp. 1446–1455, Dec. 2014, doi: 10.1093/ajh/hpu063.

[32] T. Matsuoka, M. Abe, and H. Kobayashi, ‘Iron Metabolism and Inflammatory Mediators in Patients with Renal Dysfunction’, *Int. J. Mol. Sci.*, vol. 25, no. 7, Art. no. 7, Jan. 2024, doi: 10.3390/ijms25073745.

[33] Y. Qin *et al.*, ‘Anaemia in relation to body mass index and waist circumference among Chinese women’, *Nutr. J.*, vol. 12, p. 10, Jan. 2013, doi: 10.1186/1475-2891-12-10.

[34] R. Patil and U. Raghuwanshi, ‘Serum Protein, Albumin, Globulin Levels, and A/G Ratio in HIV Positive Patients’, *Biomed. Pharmacol. J.*, vol. 2, no. 2, pp. 321–325, Feb. 2015.
[35] G. Tsirpanlis *et al.*, ‘Serum albumin: a late-reacting negative acute-phase protein in clinically evident inflammation in dialysis patients’, *Nephrol. Dial. Transplant.*, vol. 20, no. 3, pp. 658–660, Mar. 2005, doi: 10.1093/ndt/gfh663.
[36] Y. Berhane, D. Haile, and T. Tolessa, ‘Anaemia in HIV/AIDS Patients on Antiretroviral Treatment at Ayder Specialized Hospital, Mekele, Ethiopia: A Case-Control Study’, *J. Blood Med.*, vol. 11, pp. 379–387, 2020, doi: 10.2147/JBM.S275467.
[37] B. N. Harding *et al.*, ‘Antiretroviral drug class and anaemia risk in the current treatment era among people living with HIV in the USA: a clinical cohort study’, *BMJ Open*, vol. 10, no. 3, p. e031487, Mar. 2020, doi: 10.1136/bmjopen-2019-031487.

Supporting information

S1 File: the strobe checklist

S2 File: Minimal dataset

Figure

Click here to access/download;Figure;Fig. 1 sex diff (2).tif

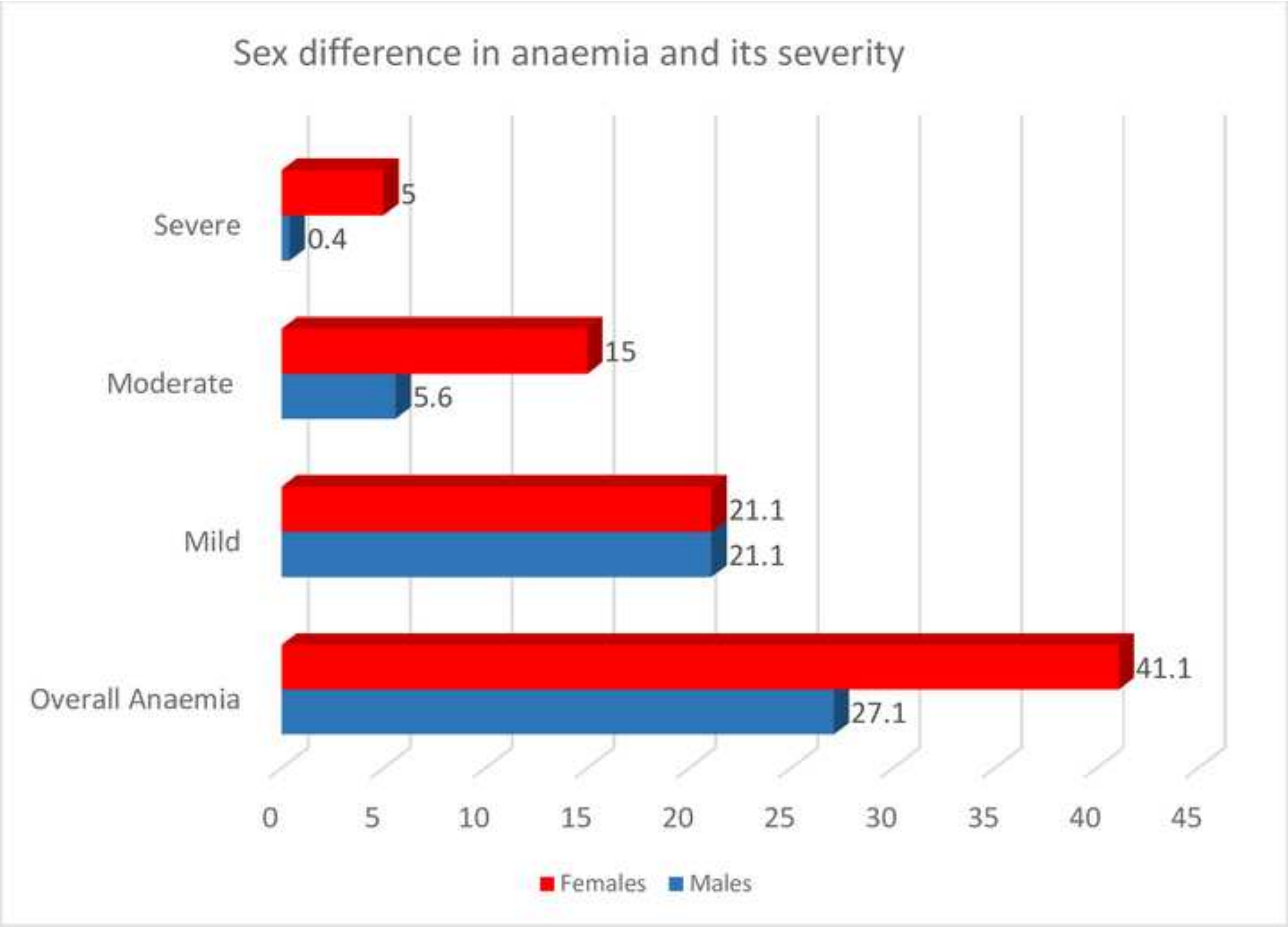

Figure

Click here to access/download;Figure;Fig 2 sex diff.tif

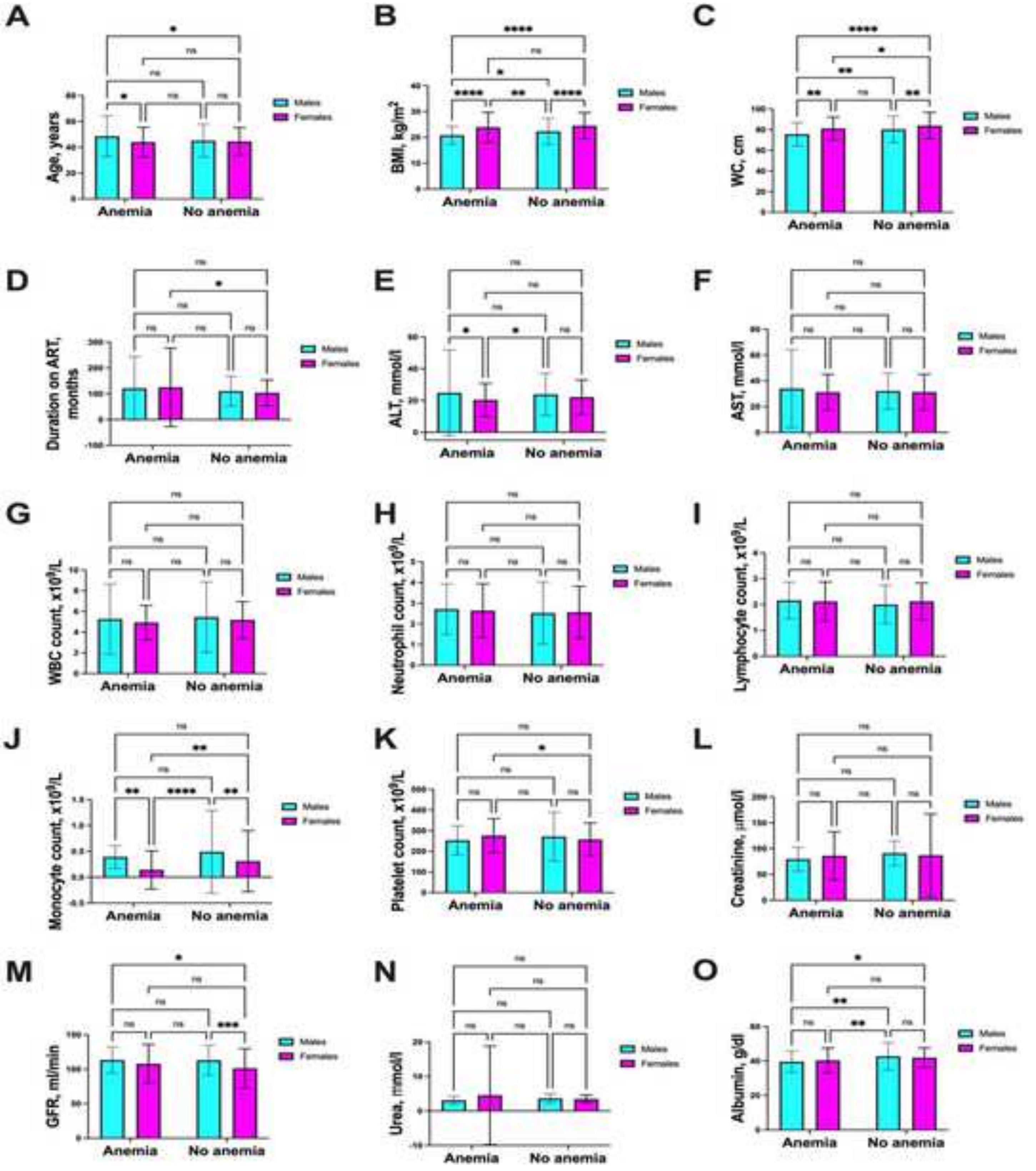

Click here to access/download
Supporting Information
S1 File. strobe checklist.pdf

Click here to access/download
Supporting Information
S2 File. Minimal Dataset .xlsx
