# Supplementary material for: Sex differences in the risk profiles for anemia in people living with HIV, A cross sectional study
Source: PLoS One. 2025 Mar 10;20(3):e0319611. doi: 10.1371/journal.pone.0319611 (PMC11892841; doi:10.1371/journal.pone.0319611)
Supplement: Attachment — Submitted filename: PLOS ONE MANUSCRIPT JAN 2025.pdf [file pone.0319611.s004.pdf]

<u>Reviewer’s comments</u>

Background:

The section highlights the necessity for regular anaemia screening among people living with HIV (PLWH) and points out the insufficient research addressing sex differences in anaemia risk profiles.

1. Please add little bit more detail on discussion of the relationship between HIV and anaemia.
2. Provide more information regarding the significance of investigating sex differences in anaemia among PLWH to strengthen the study's rationale.

Methods:
The procedures for determining sample size and statistical analysis are clearly outlined, promoting transparency in the methodology.

a) Offer a brief overview of the specific questionnaires used and how data was collected, ensuring greater transparency about the methods utilized in the study
b) The eligibility criteria are defined well; however, further elaboration on the rationale behind specific inclusions and exclusions would enhance clarity. Please clarify why the study includes only adults and the reasoning for requiring a minimum treatment duration of six months, as these explanations would justify the decisions made.

Results:
Several factors, including waist circumference and NNRTI regimens, were found to be significantly associated with anaemia, particularly in female participants.

a) Add clarity in some statistical interpretations to reinforce findings and their practical significance in clinical management.
b) For an example the following statement: Similarly, males with anaemia exhibited a lower waist circumference than females, was particularly more pronounced in those with anaemia.

Discussion:
The findings emphasize the need for sex-specific interventions, including enhanced nutritional support and a review of ART regimens.

a) Please note that Sub-Saharan Africa consists of West Africa (approximately 15 countries), East Africa (approximately 13 countries), Central Africa (approximately 8 countries), and Southern Africa (approximately 8 countries). This study focuses on a small area within one of the eight Southern African countries; therefore, it would be inaccurate to describe people living with HIV (PLWH) in this study as representative of the entire Sub-Saharan Africa population. Such generalisation is misleading to the readers outside within and outside the Sub-Saharan region.
b) Mention or outline specific areas for future research, such as larger sample sizes or studies examining additional contextual factors affecting anaemia.
