# Supplementary material for: Sex differences in the risk profiles for anemia in people living with HIV, A cross sectional study
Source: PLoS One. 2025 Mar 10;20(3):e0319611. doi: 10.1371/journal.pone.0319611 (PMC11892841; doi:10.1371/journal.pone.0319611)
Supplement: Attachment — Submitted filename: Responses to reviewers.docx [file pone.0319611.s005.docx]

**Point-by-Point Response to Reviewer Comments**

**REVIEWER 1**

**Response to Reviewer’s Comments**

We greatly appreciate the positive feedback and the recognition of the importance of the findings in our manuscript. We have carefully considered all review comments and appropriately addressed each of them in the revised manuscript

**Spelling and Language Consistency:**

- We have addressed the spelling mistakes throughout the manuscript and ensured consistency in language usage and revised incorrect statements.

**Discussion of Findings:**

- We have expanded the discussion of all findings, particularly focusing on the unusual results, as suggested (See line 316 to 319). This includes a broader exploration of why certain findings, may be unusual and how they fit within the broader context of our study population ( line 331 to 337).

**Rates of Anaemia in People Without HIV:**

- While not a requirement, we acknowledge the suggestion to include data on anaemia and associated factors in the general population (without HIV). While this was not part of the current study design, we included a note in the discussion (line 367 to 369) on the potential value of future studies comparing anaemia rates between PLWH.

**REVIEWER 2**

**Background:**

1. *Comment:* Add more detail on the relationship between HIV and anaemia.
   - **Response:** We expanded the discussion on HIV and anaemia (line 77 to 86), highlighting HIV-induced chronic inflammation and ART-related factors contributing to anaemia.
2. *Comment:* Provide more information on investigating sex differences in anaemia among PLWH.
   - **Response:** We included a detailed explanation of the importance of sex differences (line 92 to 97).

**Methods:** a) *Comment:* Brief overview of questionnaires and data collection.

- **Response:** We clarified the data collection methods (line 123 to 128), mentioning the use of standardized questionnaires and clinical data from ART registries.

b) *Comment:* Clarify rationale for including only adults and minimum treatment duration.

- **Response:** We clarified that adults were included due to physiological differences (line 111 to 116) and the six-month ART duration ensures stable treatment effects.

**Results:** a) *Comment:* Add clarity in statistical interpretations.

- **Response:** We revised and added further discussion (line 331 to 337), emphasizing the clinical significance of waist circumference and ART regimen associations with anaemia.

b) *Comment:* Clarify the comparison between males and females regarding waist circumference.

- **Response:** The sentence was revised for clarity (line 220 to 223).

**Discussion:** a) *Comment:* Avoid generalizing to all of Sub-Saharan Africa.

- **Response:** We revised the manuscript to avoid generalisation to the Sub-saharan africa

b) *Comment:* Outline areas for future research.

- **Response:** Future research directions were added (line 359 to 366), suggesting larger sample sizes and further exploration of contextual factors like socio-economic status and nutrition.

We thank the reviewers for their valuable feedback, which has helped improve the manuscript.
